# Microvascular obstruction assessed by 3-tesla magnetic resonance imaging in acute myocardial infarction is correlated with plasma troponin I levels

**DOI:** 10.1186/1471-2261-14-57

**Published:** 2014-04-30

**Authors:** Karine Pernet, Fiona Ecarnot, Romain Chopard, Marie-France Seronde, Philoktimon Plastaras, Francois Schiele, Nicolas Meneveau

**Affiliations:** 1 Department of Cardiology, EA3920, University Hospital Jean Minjoz, Boulevard Fleming, Besançon 25000, France

**Keywords:** Myocardial infarction, MRI, Troponin, Microvascular obstruction

## Abstract

**Background:**

Microvascular obstruction (MVO) at the acute phase of myocardial infarction (MI) is associated with poor prognosis. We aimed to evaluate the correlation between plasma cardiac troponin I (cTnI) at the acute phase of MI and extent of no-reflow, as assessed by 3-T cardiac magnetic resonance imaging (MRI). Secondly, we defined a cut-off value for cTnI predictive of no-reflow.

**Methods:**

51 consecutive patients with no previous history of cardiovascular disease, presenting ST elevation MI within <12 h. Infarct size and extent of no-reflow were evaluated by 3-T MRI at day 5. Extent of no-reflow at 15 minutes (MVO) was correlated with cTnI at admission, 6, 12, 24, 48 and 72 hours. At 6 months, MRI was performed to evaluate the impact of MVO on LV remodeling.

**Results:**

MVO was diagnosed in 29 patients (57%). Extent of MVO was significantly correlated to peak troponin, cTnI (except admission values) and area under the curve. Using Receiver-operating characteristic (ROC) curve analysis, a cut-off cTnI value >89 ng/mL at 12 h seemed to best predict presence of early MVO (sensitivity 63%, specificity 88%). At 6 months, MVO was associated with left ventricular (LV) remodeling, resulting in higher LV volumes.

**Conclusion:**

There is a relationship between cTnI at the acute phase of AMI and extent of MVO as assessed by 3-T cardiac MRI. A cut-off cTnI value of 89 ng/mL at 12 h seems to best predict presence of MVO, which contributes to LV remodeling.

## Background

In acute ST-segment-elevation myocardial infarction (STEMI), primary percutaneous coronary intervention is the treatment of choice to restore myocardial revascularization and minimize ischemic damage to the myocardium [1]. Several studies have demonstrated that microvascular dysfunction after infarct-related artery revascularization is associated with myocardial reperfusion injury, resulting in greater infarct size [2-10], left ventricular impairment [3-5,7,11-17], recurrent MI [2-4,14], heart failure [2-4,12,14] and higher mortality [2-5,12,14,18,19]. In addition, microvascular dysfunction has a negative prognostic value, irrespective of infarct size [3]. Magnetic Resonance Imaging (MRI) is not systematically performed in routine practice, and it would thus be useful if there was a more accessible prognostic marker that could identify patients with microvascular obstruction (MVO). Cardiac troponin levels correlate well with infarct size [20] and are of prognostic value in the short and long term [21].

The aim of our study was to evaluate the relationship between plasma levels of cardiac troponin I (cTnI) and microvascular obstruction (MVO) as assessed by magnetic resonance imaging (MRI) at day 5, and to define a cut-off value for cTnI that predicts MVO.

## Methods

### Study population

This study was a prospective single-center study. Patients < 75 years old referred to our department for a first ST elevation MI (STEMI) and admitted within 12 hours of symptom onset were considered for inclusion. MI was defined by the guidelines of the joint Task Force of the European Society of Cardiology (ESC), the American College of Cardiology (ACC), the American Heart Association (AHA), and the World Heart Federation (WHF) [1,22]. MI was confirmed by detection of elevated cardiac biomarkers (at least one value above the 99^th^ percentile of the upper reference limit (URL)), together with evidence of myocardial ischemia (i.e., new ST-T changes or new left bundle branch block, or development of pathological Q waves in the ECG).

Exclusion criteria were: previous cardiovascular or pulmonary diseases, cardiogenic shock and contraindication for MRI.

The study protocol was approved by the local ethics committee (Comité de Protection des Personnes Est II, University Hospital Besancon, France) and informed consent was obtained from all enrolled patients.

### Angiographic evaluation

All patients were referred to the catheterization laboratory within the first 24 hours after admission for coronary angiogram, and received medication according to current guidelines [1].

The initial and post-procedural blood flow in the infarct-related artery was graded according to the Thrombolysis in Myocardial Infarction (TIMI) grading system [23]. A successful procedure was defined by a TIMI flow grade = 3 and residual stenosis < 20%.

### Magnetic resonance imaging

All CE-MRI studies were conducted at 3.0 field strength (Signa HD, General Electric Healthcare, Milwaukee, WI, USA) and performed in the acute phase and repeated at 6 months. Left ventricular function was assessed by ECG-gated cine steady-state free precession (SSFP) breath-hold sequences in the two-chamber and four-chamber views as well as in the short cardiac axis from base to apex (30 phases per cardiac cycle; repetition time 3.5 ms, echo time 1.2 ms, flip angle 45°, typical voxel size 1.92 × 1.25 × 8.0 mm).

Contrast-enhancement imaging was performed at 3 and 15 min with a breath-hold ECG-gated T1-weighted sequence after the injection of a bolus of gadolinium (Dota-Gd; Guerbet, Roissy, France) at a single dose of 0.1 mmol/kg (TE = MinFull/field of view 440 mm/TI = optimised to obtain an optimal myocardial nulling/matrix 256 × 224, interpolated 256 × 256/slice thickness = 8 mm/gap = 1 mm). The number and position of slices were the same as used for functional imaging.

Image analysis was performed in a blind fashion by two operators using an off-line dedicated workstation (General Electric Healthcare, Milwaukee, WI, USA). Left ventricular (LV) ejection fraction (LVEF), end-diastolic (LVEDVI) and end–systolic volume index (LVESVI) and mass were calculated from SSFP short-axis views. MVO was assessed on the initial CE-MRI performed during in-hospital stay. Infarct size was assessed from the same initial CE-MRI and at 6 months follow-up. Infarct size and MVO (if present) were manually traced from the contrast-enhancement short-axis images [24]. Myocardial regions was considered infarcted if the infarct size signal intensity was >2 SDs above the remote myocardium. MVO was defined as a dark zone within the infarcted segments, usually located in the subendocardium. MVO and infarct size are expressed as a percentage of LV mass assuming 1.05 g/ml as the specific gravity of myocardium.

### Enzyme measurement

Serum levels of troponin I (TnI), creatine kinase (CK) and creatine kinase isoenzyme myocardial and brain (CK-MB) were determined on admission, and 6, 12, 24, and 48 hours after onset of MI. cTnI was measured quantitatively using sandwich immunoassay (Dimension RXL max, SIEMENS, Germany). The lower limit of detection for this assay was 0.04 ng/ml. A value of cTnl > 0.15 ng/ml was considered indicative of myocardial necrosis for the purposes of clinical care. The peak level was determined as the highest value.

### Statistical analysis

Quantitative continuous variables are expressed as mean ± standard deviation, and qualitative variables as number and percent. The Student t test and Chi square or Fisher’s exact tests were used for comparisons, as appropriate. We calculated the relative change at 6 months from baseline in LV end-diastolic volume index, LV end-systolic volume index, and LV ejection fraction. The model used to take initial infarct size into account was the calculation of relative change, normalized for baseline infarct size. The correlations between serum enzyme levels and MVO at day 5 were assessed using the Spearman rank test. A receiver-operating characteristic (ROC) curve was used to define the threshold value of troponin levels predictive of MVO at day 5. A p-value <0.05 was considered statistically significant. All analyses were performed using SAS version 9.2 (SAS Institute, Cary, NC, USA).

## Results

### Patient characteristics

Between 2006 and 2008, 51 patients were enrolled in the study. Mean age was 54 years; there were 9 women and 42 men. Baseline and angiographic characteristics of the study population are shown in Table 1. Eighteen patients (35%) had anterior MI location. All patients underwent coronary angiogram and angioplasty within the first 24 hours. Reperfusion therapy was initiated 5.3 ± 3.2 hours after the onset of symptoms, by primary PCI in 37 patients (73%) and thrombolysis in 14 patients (27%). In patients who underwent thrombolysis, rescue PCI was performed in 6 patients (12%) and the remaining 8 underwent systematic PCI within the first 24 hours after reperfusion.

**Table 1:** Baseline and angiographic characteristics

	**All patients**	**Pts MVO**^**neg**^	**Pts MVO**^**pos**^	**P**
	**N = 51**	**N = 22 (43%)**	**N = 29 (57%)**	
**Age - years**	53.6 ± 9.9	56 ± 10	52.6 ± 9.4	0.27
**Female (%)**	9 (18)	4 (19)	5 (17.5)	0.93
**Arterial hypertension (%)**	18 (35)	7 (32)	11(38)	0.65
**Diabetes mellitus (%)**	11 (22)	5 (23)	6 (21)	0.86
**Family history of CAD (%)**	15 (29)	7 (32)	8 (27)	0.74
**Current smoking**	42 (82)	18 (82)	24 (83)	0.93
**Hyperlipidemia (%)**	21 (41)	9 (41)	12 (41)	0.97
**Body mass index ≥30Kg/m**^**2**^**(%)**	9 (18)	3 (14)	6 (20.6)	0.51
**One vessel disease (%)**	28 (55)	13 (59)	15 (52)	0.60
**Multivessel disease (%)**	16 (31)	7 (32)	9 (31)	0.95
**Stenosis < 50% (%)**	7 (14)	2 (9)	5 (17)	0.40
**Anterior location (%)**	18 (35)	9 (41)	9 (31)	0.46
**Time from symptom onset to 1**^**st **^**reperfusion therapy – hours**	5.3 ± 3.2	4.7 ± 2.9	5.9 ± 3.7	0.21
**Fibrinolysis (%)**	14 (27)	5 (23)	9 (31)	0.73
**Primary PCI (%)**	37 (73)	17 (77)	20 (69)	
**Rescue PCI (%)**	6 (12)	2 (9)	4 (14)	0.94
**Aspirin**	51 (100)	22 (100)	29 (100)	1
**Clopidogrel loading dose (300/600 mg)**	34/17	14/8	20/9	0.69
**GP IIb-IIIa inhibitors (%)**	22 (43)	8 (36)	14(48)	0.39
**TnI peak (ng/mL) (range)**	81 ± 71	53.6 (13–225)	118.5 (22–585)	0.0006
**CK peak, U/L (range)**	1985 ± 1522	1825 (63–9580)	2663 (160–9930)	0.006
**CK-MB peak, U/L (range)**	124 ± 132	145.6 (9–940)	189.1 (9–760)	0.09

Pts MVO^neg^ = patients without microvascular obstruction; Pts MVO^pos^ = patients with microvascular obstruction; CAD = coronary artery disease; PCI = percutaneous coronary intervention; TnI = troponin I; CK = creatine kinase; CK-MB = creatine kinase isoenzyme myocardial and brain.

**Figure 1:**
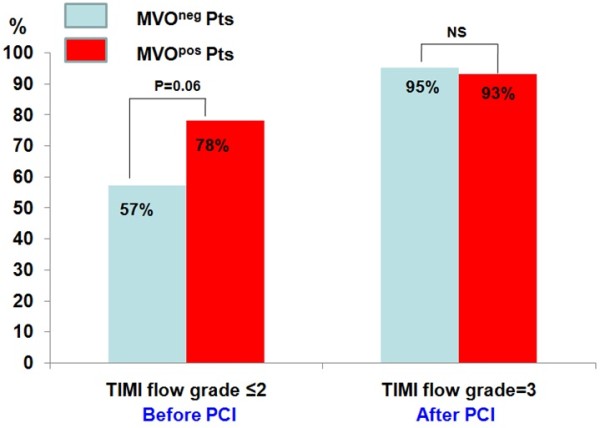
**TIMI flow grade before and after percutaneous coronary intervention.** TIMI = thrombolysis in myocardial infarction; PCI = percutaneous coronary intervention; MVO^neg^ = patients without microvascular obstruction; MVO^pos^ = patients with microvascular obstruction.

Twenty-nine patients (57%) had MVO (MVO^pos^ patients) diagnosed by MRI and 22 (43%) had no MVO (MVO^neg^ patients). MVO^pos^ and MVO^neg^ patients were comparable as regards baseline characteristics.The percentage of patients with TIMI flow grade ≤2 before PCI was significantly higher in the MVO ^pos^ group. After PCI, the rate of TIMI flow grade 3 was comparable in both groups (Figure 1).

During follow-up, there were 0 deaths, and 4 revascularisations (1 for recurrent MI due to stent thrombosis at 12 days and 3 on arteries other than the infarct-related artery).

### Magnetic resonance imaging

MRI was performed at 5.7 ± 2.7 days after symptom onset and at 6 ± 1 months follow-up. MRI was not performed in 6 patients at 6 months due to patient refusal, or intervention (PCI or CABG) during the follow-up period.

At day 5, the extent of MVO was 7.3 ± 3.7%. Infarct size was 23.5 ± 8.4% in MVO^pos^ patients versus 12.7 ± 9.4% in MVO^neg^ patients (p = 0.0001). There was a significant correlation between the extent of MVO at day 5 and the infarct size at day 5 (r = 0.61, p < 0.0001). Similarly, we observed a significant correlation between extent of MVO and time to reperfusion (r = 0.43, p = 0.004).

**Figure 2:**
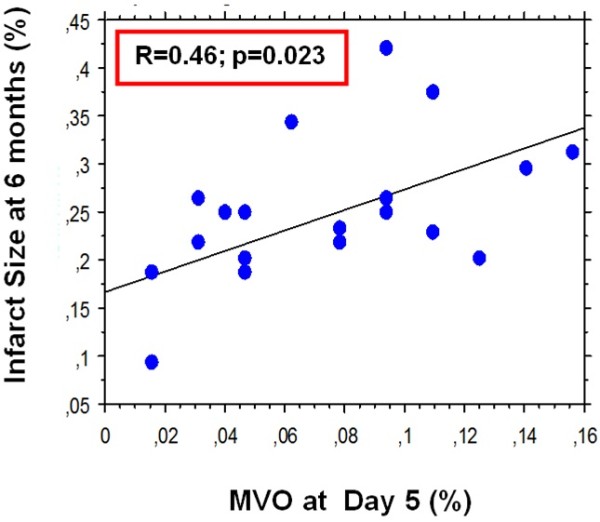
**Relationship between MVO at Day 5 and infarct size at 6 months.** MVO = microvascular obstruction; MVO^pos^ **=** patients with microvascular obstruction.

**Figure 3:**
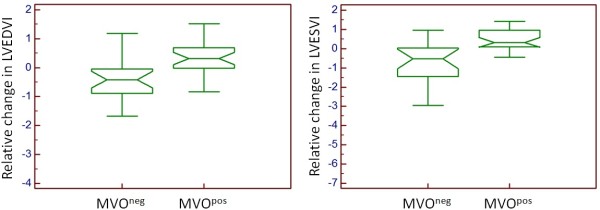
**Relative change at 6 months from baseline in left ventricular end-diastolic volume index (LVEDVI) and left ventricular end-systolic volume index (LVESVI) normalized for baseline infarct size.** MVO^neg^ = patients without microvascular obstruction; MVO^pos^ = patients with microvascular obstruction.

At 6 months follow-up, a significant positive correlation was observed among MVO^pos^ patients between MVO at day 5 and infarct size at 6 months (Figure 2). In MVO^pos^ patients, the relative change in LV end-diastolic volume index (LVEDVI) and in LV end-systolic volume index (LVESVI) was significantly greater than in MVO^neg^ patients (p = 0.002 for LVEDVI, p = 0.004 for LVESVI) (Figure 3). There was no significant difference in relative change in LV ejection fraction (LVEF).

### Enzyme measurement

Peak troponin and peak CK were significantly higher in MVO^pos^ patients (respectively p = 0.0006 and p = 0.006) (Table 1).

There was no significant difference in terms of peak troponin values between patients with TIMI flow grade 3 and those with TIMI flow ≤2 after revascularisation (peak troponin 75 ± 58 vs 83 ± 65, TIMI 3 vs ≤2 respectively, p = 0.65).

#### Correlation between the extent of MVO and troponin I levels

**Figure 4:**
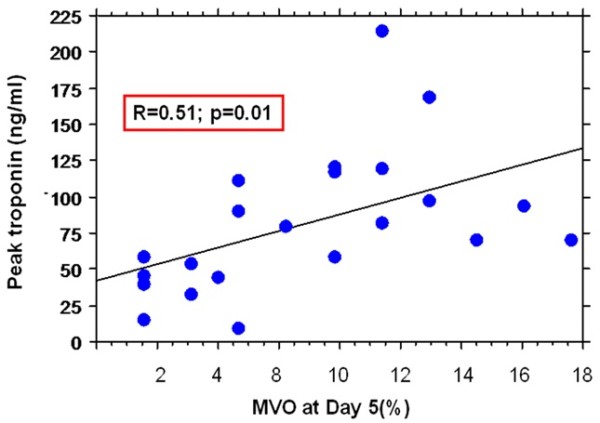
**Relationship between MVO and Peak troponin.** MVO = microvascular obstruction.

The extent of MVO at day 5 was significantly correlated with peak troponin (Figure 4) and troponin I levels at 6, 12, 24, 48 and 72 hours (Table 2). Infarct size was significantly correlated with peak troponin (p < 0.0001) and the different cTnI levels (p < 0.001), except cTnI at admission.

**Table 2:** Correlation between the extent of MVO and troponin I levels

	**MVO (N = 29)**	
	**r**	**p**
Troponin peak	0.51	0.01
AUC troponin	0.58	0.01
Troponin on admission	0.11	0.59
Troponin at 6H	0.57	0.01
Troponin at 12H	0.67	<0.0001
Troponin at 24H	0.46	0.02
Troponin at 48H	0.57	0.01
Troponin at 72H	0.42	0.04

MVO = microvascular obstruction; AUC = area under the curve; H = hours.

#### Relationship between the presence of MVO and troponin I levels

**Figure 5:**
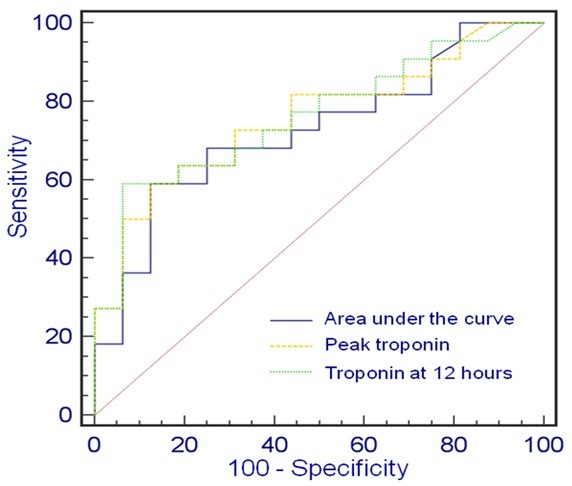
**Cut-off value of cTnI predictive of MVO.** ROC curves established for: AUC troponin (AUC _ROC_ 0.726, 95% CI 0.56-0.86). Peak troponin (AUC_ROC_ 0.757, 95% CI 0.59-0.88). Troponin at 12 hours (AUC_ROC_ 0.763, 95% CI 0.6-0.88) cTnI = cardiac troponin I; MVO = microvascular obstruction; ROC = receiver operating characteristic; AUC = area under the curve.

Peak troponin and all cTnI levels except cTnI at admission were significant predictors of MVO (Table 3). The Area under the curve (ROC) varied from 0.68 for troponin at 72 h to 0.766 for troponin at 12 h. Sensitivity varied from 56% for troponin at 48 h to 74% for troponin at 6 h. Specificity ranged from 65% for troponin at 6 h and 72 h to 88% for troponin at 12 h, 48 h and the AUC troponin. Using ROC curve analysis, a cTnI level > 89 ng/ml at 12 hours was found to be the best cut-off value to predict MVO with a sensitivity of 63% and a specificity of 88% (p < 0.0002) (Figure 5).

**Table 3:** Cut-off value of cTnI predictive of MVO

	**Cut-off value (ng/ml)**	**Se (%)**	**Sp (%)**	**AUC ROC**	**p**
Troponin at H6	44.6	74	65	0.729	0.0025
Troponin at H12	89	63	88	0.766	0.0002
Troponin at H24	27.4	65	81	0.703	0.0116
Troponin at H48	16.35	56	88	0.724	0.0041
Troponin at H72	7.9	71	65	0.68	0.0308
Peak troponin	79.9	63	82	0.761	0.0003
AUC Troponin	275.3	59	88	0.726	0.0057

Se **=** sensitivity; Sp = specificity; AUC = area under the curve; ROC = receiver operating characteristic; H = hours.

## Discussion

We aimed to find an accessible prognostic marker that could identify patients with microvascular dysfunction after reperfusion at the acute phase of myocardial infarction. Our data demonstrated that troponin levels (except troponin at admission), and particularly peak troponin, were correlated with the extent of MVO on MRI at day 5. A troponin I level > 89 ng/ml at 12 hours was found to be the best cut-off value to predict MVO, with a sensitivity of 63% and a specificity of 88%.

The phenomenon of no-reflow [25,26] or microvascular obstruction is due to microembolization of atherosclerotic debris or thrombotic material, intimal oedema, tissue haemorrhage and/or leukocyte sticking [8,27], and is diagnosed well by MRI [3,6,13,28]. In addition, besides microvascular obstruction, intra-myocardial hemorrhage and microvascular destruction have also been shown to play an important role in this setting [29]. At the acute phase of MI, the successful restoration of epicardial coronary artery patency does not always lead to adequate reperfusion at the microvascular level. In our study, although 93 to 95% of the patients had a TIMI flow grade =3 after PCI at 24 h, MVO was diagnosed by MRI in 57% of cases at day 5. There was no significant difference in peak troponin between patients with TIMI flow grade 3 and those with TIMI flow ≤2 after revascularisation. Several studies have shown that microvascular dysfunction after infarct-related artery revascularization is associated with myocardial reperfusion injury, resulting in greater infarct size [2-10], left ventricular impairment [3-5,7,11-17], recurrent MI [2-4,14], heart failure [2-4,12,14] and higher mortality [2-5,12,14,18,19]. In patients with MVO, mortality has been reported to increase by 75% at 1 month [18], 67% at 1 year [5] and 50% at 5 years [19]. In our study, MVO at day 5 was associated with LV remodelling at 6 months, as reflected by significantly increased left ventricular volumes assessed on MRI. These results are comparable to those of Orn et al. [7] at 1 year, and those of Nijveldt et al. [13] at 4 months, using the MRI technique.

This is strong evidence that both serial and single-point measurements of troponin correlate well with infarct size [8,9,20]. In routine practice, plasma levels of troponin I are used by the clinician to estimate the extent of necrosis. In our study, infarct size was significantly correlated with peak troponin and with the serial troponin I levels, except for troponin values at admission. To date, only 3 studies have investigated the relationship between MVO as assessed by MRI and serial troponin measurements, and the optimal timing for a single measurement. Our results confirm those of the literature. In the study of Neizel et al. [8], 61 patients with STEMI <24 h reperfused by fibrinolysis or PCI, underwent MRI within 4 ± 1 days and serial troponin T measurements at admission and after 24, 48, 72 and 96 hours. They found that a troponin T level > 2.52 μg/l at 24 h was a predictor for MVO with a sensitivity of 100% and a specificity of 80%. In this study, Neizel et al. used troponin T levels, and no troponin measurements were performed between admission and 24 h. Furthermore, MVO was evaluated on earlier delayed-enhancement imaging after contrast injection. Younger and al [9] enrolled 93 patients who underwent MRI on average 3.7 ± 1.4 days after medical treatment for acute ST elevation or non-ST elevation myocardial infarction. Serum troponin I concentrations were sampled at 12 h and 72 h only after admission. They found a close relationship between a single measurement of troponin I at 72 h and the extent of MVO. The early and complete reperfusion of the infarct-related artery accelerates the appearance of cardiac enzymes in the first hours after reperfusion therapy. This wash-out phenomenon is characterized by a steeper rise and an earlier peak after successful restoration of anterograde coronary blood flow. These changes are more pronounced after mechanical reperfusion, which was used in our study, than after thrombolytic therapy [8]. Therefore, the almost exclusive reliance on thrombolytic therapy in the study of Younger and al [9] may explain the more delayed troponin release observed. In study by Mayr and al [10], MRI was performed in 118 patients within 8 days after successful interventional reperfusion of STEMI. Cardiac troponin T concentrations were measured at least 3 times during the first 24 h after admission, and subsequently on days 1, 2, 3 and 4. These authors demonstrated that a peak troponin > 4.7 μg/l was predictive of MVO with a sensitivity of 78% and a specificity of 83%.

One of the advantages of the present study is the large number of repeat troponin measures, at relatively short time intervals, which allowed more accurate determination of a threshold value that could predict MVO at the acute phase of infarction.

It has previously been shown that a longer duration of ischemia is associated with more pronounced impairment of myocardial perfusion and consequently, infarct size [30]. In our study, we observed a correlation between the extent of MVO and the time to reperfusion. Thus, the relation between troponin and the presence of MVO should be interpreted taking into account the time to reperfusion, since restoration of TIMI flow grade 3 by angioplasty does not necessarily guarantee the normalization of myocardial perfusion [31].

Newton et al. previously studied MVO on MRI in the context of non-ST elevation MI, and observed that MVO was correlated with infarct size and troponin levels. Thus, it would appear that the results observed in the context of STEMI are also applicable in NSTEMI [32].

### Study limitations

This is a single-center study, with a relatively small population of highly selected patients. Nonetheless, the selection of the population made it possible to independently analyse MVO as evaluated by MRI in the absence of other cardiovascular comorbidities. Secondly, we did not evaluate blush grade, which corresponds to the angiographic evaluation of microcirculation after revascularisation. Our study focused on MVO at 5 days, which is a different phenomenon, and shown to be an independent prognostic factor in acute MI. These results merit further confirmation in a larger, prospective study. Thirdly, reperfusion strategy (primary PCI or thrombolysis) may possibly influence the occurrence of MVO. In particular, it has been shown that rescue PCI is associated with an increased thrombotic risk in this setting. However, in our study, only 6 (12%) patients underwent rescue PCI. This likely did not impact on the relation observed between MVO and peak troponin, as there was no significant difference between groups (Table 1). Fourthly, MRI could not be performed in 6 patients (11.7%) at 6 months. However, this does not impact the interpretation of the primary endpoint evaluated at 5 days.

## Conclusion

Our results suggest a correlation between plasma levels of cardiac troponin I at the acute phase of AMI, and the extent of MVO as assessed by 3-T cardiac magnetic resonance imaging. The extent of MVO at 5 days post-STEMI is correlated with infarct size, and negatively impacts on LV remodeling at 6 months. A cut-off value of 89 ng/mL for cTnI at 12 hours seems to best predict the presence of early MVO with high sensitivity and specificity. Troponin measurement is an easily accessible prognostic marker that could help identify patients with MVO and unfavorable prognosis.

## Competing interests

The authors declare that they have no competing interests.

## Authors’ contributions

Study conception and design: KP, MFS, NM. Acquisition of data: KP, RC, MFS, PP, FS, NM. Analysis and interpretation of data: FE, RC, PP, FS, NM. Drafting of the manuscript and critical revision: All. All authors read and approved the final version of the manuscript to be published. All authors agree to be accountable for all aspects of the work in ensuring that questions related to the accuracy or integrity of any part of the work are appropriately investigated and resolved.

## Pre-publication history

The pre-publication history for this paper can be accessed here:

http://www.biomedcentral.com/1471-2261/14/57/prepub
